# Immunoreactive cutaneous sporotrichosis^⋆^^⋆⋆^

**DOI:** 10.1016/j.abd.2019.11.015

**Published:** 2020-08-16

**Authors:** Gustavo de Sá Menezes Carvalho, John Verrinder Veasey

**Affiliations:** Dermatology Clinic, Irmandade da Santa Casa de Misericórdia de São Paulo, São Paulo, SP, Brazil

**Keywords:** Erythema nodosum, Mycoses, Sporotrichosis, Zoonoses

## Abstract

*Sporothrix* spp. infection can occur through the inoculation of the organism in the skin through direct contact with the soil (sapronotic infection), through contact with animals, such as infected cats and dogs (zoonotic infection), or less frequently *via* inhalation. With a subacute or chronic evolution, approximately 80% of patients affected by the disease present with the lymphocutaneous form; episodes associated with a hypersensitivity reaction are rare. The authors report the case of a 12-year-old child with immunoreactive sporotrichosis manifested clinically as erythema nodosum lesions in the lower limbs, associated with an ulcerated lesion in the left arm.

Sporotrichosis is an infection that affects humans and animals, with a typically subacute or chronic evolution, caused by the dimorphic fungus *Sporothrix* spp.^1^, ^2^ Approximately 80% of patients affected by the disease present with the lymphocutaneous form^2^; cases of hypersensitivity reaction to *Sporothrix* spp. are rare, with few reports in the literature.^2^ The authors report a case of a 12-year-old girl who presented a single ulcerated lesion on the left arm with a raised erythematous edge and granular bottom, measuring 1.5 cm in diameter (Fig. 1). One month after the onset of the condition, painful erythematous nodules were observed on the lower limbs, more palpable than visible, accompanied by feverish episodes, without any use of medication or other infectious complaints in the period (Fig. 2). The chest radiography did not reveal any abnormalities, and no skin reaction was observed in the tuberculin skin test. Samples of the ulcerated lesion were collected; the direct microscopy examination (DME) did not show fungal structures or amastigote forms of *Leishmania* spp., the polymerase chain reaction for leishmaniasis was negative, and *Sporothrix* spp. was observed in the culture for fungi (Figure 3, Figure 4). The histopathological examination of the ulcerated lesion showed inflammation in the hypodermis with the formation of granulomas, but no fungi were observed with Grocott staining. The diagnosis of immunoreactive cutaneous sporotrichosis was established due to the appearance of erythema nodosum in association with the fungal infection, with no other evident cause.

**Figure 1:**
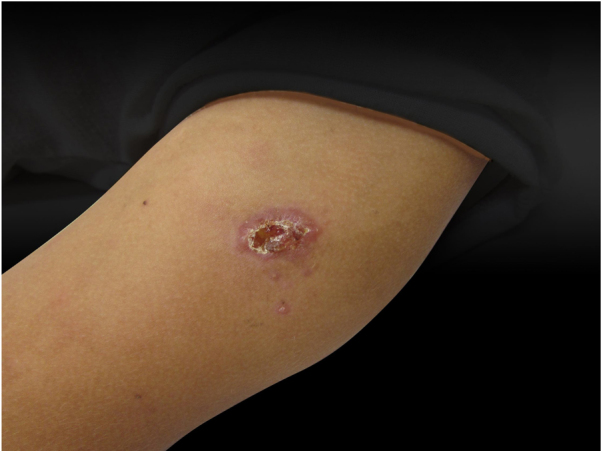
Dermatosis located in the upper portion of the left arm, characterized by an erythematous ulcer with well-defined edges and a granular bottom measuring approximately 1.5 cm in diameter.

**Figure 2:**
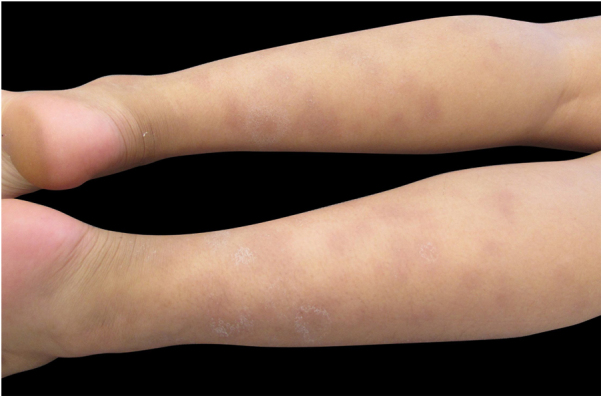
Dermatosis located on the lower limbs, characterized by erythematous and violet nodules of different sizes, more palpable than visible.

**Figure 3:**
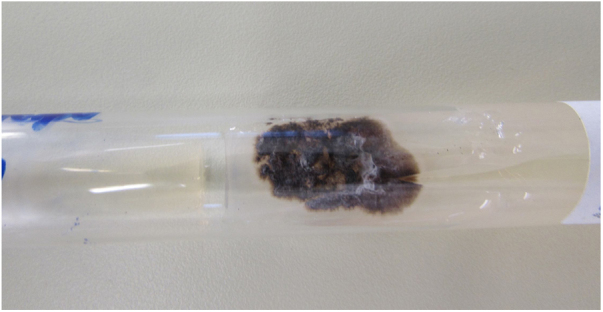
Fungal culture at 25 °C showing a blackish filamentous colony with whitish areas. Colony growth after seven days.

**Figure 4:**
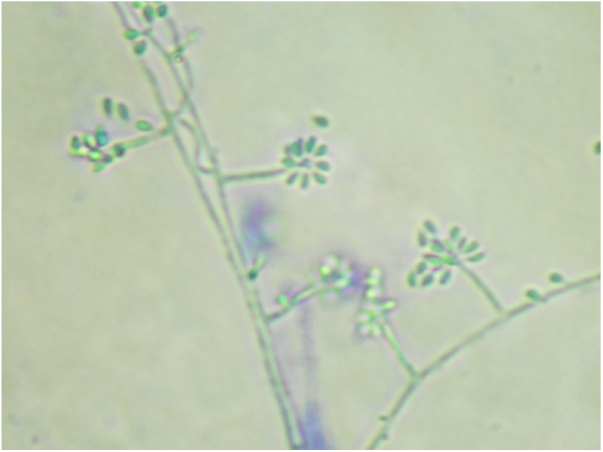
Colony micromorphology at 25 °C, showing septate hyaline hyphae, conidiophores that originate primary hyaline conidia in a “daisy” arrangement (cotton blue, ×400).

In Brazil, sporotrichosis has been an emerging zoonosis for the last 20 years. With the advent of molecular biology techniques, it has been shown that the classic agent *Sporothrix* spp. consists of a group of species among which *S. brasiliensis*, *S. schenckii*, *S. globosa*, and *S. luriei* stand out as human pathogens.^3^ With the epidemic of zoonotic sporotrichosis, clinical forms hitherto uncommon are being described, such as hypersensitivity reactions,^4^, ^5^, ^6^ which can present as erythema multiforme, Sweet's syndrome, or erythema nodosum. Patients with such presentations have an exacerbated immune response to the fungus.^1^, ^4^, ^5^ In the present case, it is interesting to observe that such immunological competence may have prevented dissemination of the pathogen through the lymphatic pathway, avoiding the most common lymphocutaneous manifestation, and exhibiting a localized cutaneous clinical presentation, evidenced at first by the patient. This clinical form is of difficult differentiation from ulcerated lesions caused by other tropical dermatoses such as tuberculosis, paracoccidioidomycosis, and leishmaniasis, also endemic in the country.

The gold standard for the diagnosis of sporotrichosis is the isolation of the fungal agent from clinical samples. Elongated yeast structures are rarely seen on direct examination.^2^, ^3^, ^7^ Isolation of the agent in culture media has superior sensitivity and specificity. The colonies present a blackish color and a membranous appearance, sometimes showing whitish areas (Fig. 3); in the microculture, septate hyaline hyphae and conidiophores that originate primary hyaline conidia in a “daisy” arrangement are observed. Similar to the direct examination, most of the time histopathology has low sensitivity, due to the scarcity of fungal elements in the sample.^7^

The report of this case of sporotrichosis illustrates a rare presentation of this condition, aiming to contribute to early diagnosis and treatment, reducing the chronicity and morbidity of the disease.

## Financial support

None declared.

## Authors’ contributions

Gustavo de Sá Menezes Carvalho: Elaboration and writing of the manuscript; obtaining, analyzing, and interpreting the data.

John Verrinder Veasey: Approval of the final version of the manuscript; conception and planning of the study; elaboration and writing of the manuscript; obtaining, analyzing, and interpreting the data; effective participation in research orientation, critical review of the literature, critical review of the manuscript.

## Conflicts of interest

None declared.
